# Dissecting Uricase Immunogenicity: Unveiling the Role of Quaternary Epitopes through In Silico Analysis: Quaternary Epitope Insights in Uricase Immunogenicity

**DOI:** 10.31661/gmj.v14i.3634

**Published:** 2025-01-29

**Authors:** Mohammad Reza Rahbar, Navid Nezafat, Mohammad Hossein Morowvat, Amir Savardashtaki, Mohammad Bagher Ghoshoon, Kamran Mehrabani-Zeinabad, Younes Ghasemi

**Affiliations:** ^1^ Pharmaceutical Sciences Research Center, Shiraz University of Medical Sciences, Shiraz, Iran; ^2^ Department of Pharmaceutical Biotechnology, School of Pharmacy, Shiraz University of Medical Sciences, Shiraz, Iran; ^3^ Department of Medical Biotechnology, School of Advanced Medical Sciences and Technologies, Shiraz University of Medical Sciences, Shiraz, Iran; ^4^ Infertility Research Center, Shiraz University of Medical Sciences, Shiraz, Iran; ^5^ Department of Biostatistics, Faculty of Medicine, Shiraz University of Medical Sciences, Shiraz, Iran

**Keywords:** Immunogenicity, Quaternary Epitopes, Epitope Mapping, In Silico Analysis, Computational Immunology, Therapeutic Proteins

## Abstract

**Background:**

Protein-based therapeutics offer remarkable precision and effectiveness, yet their immunogenic potential remains a significant challenge. Uricase, an enzyme used to treat hyperuricemia, is no exception, often eliciting immune responses due to its non-human origins and repeated administration requirements. Understanding the immunogenic mechanisms at play is crucial for enhancing therapeutic efficacy.

**Materials and Methods:**

This in silico study investigates the immunogenic landscape of uricase, focusing on the identification of linear, conformational, and the underexplored quaternary epitopes. Using a comprehensive approach, we analyzed multiple uricase variants through structural alignments, epitope prediction algorithms, and network-based residue interaction models. Predictive tools, including BepiPred, DiscoTope, and SEMA, were employed to identify epitope regions, with a novel focus on quaternary epitopes formed by inter-chain interactions.

**Results:**

Our analysis reveals conserved structural motifs across uricase variants, with linear and conformational epitopes localized in similar regions. The groundbreaking identification of quaternary epitopes—epitopes formed through interactions between protein chains—provides a novel insight into uricase immunogenicity. These epitopes, located in structurally prominent regions, likely play a critical role in the immune response to uricase.

**Conclusion:**

This study marks a significant advance in understanding uricase immunogenicity, introducing quaternary epitopes as pivotal factors in immune recognition. The findings open new avenues for designing uricase variants with reduced immunogenicity, offering potential improvements in therapeutic strategies for hyperuricemia management.

## Introduction

Protein-based therapeutics have garnered significant attention due to their high specificity and efficacy, even at low concentrations, making them superior to small molecule drugs in many therapeutic applications [1][2]. However, a major limitation of protein drugs is their potential to trigger immune responses, primarily through the elicitation of anti-drug antibodies (ADAs) [3][4]. These ADAs can accelerate drug clearance from the bloodstream, altering the pharmacokinetics and pharmacodynamics of the drug, which often leads to treatment failure or, in severe cases, adverse reactions [5][6].

One such therapeutic protein facing immunogenic challenges is uricase—an enzyme used to regulate uric acid levels in patients suffering from hyperuricemia [7][8][9]. Uricase plays a critical role in purine metabolism by converting uric acid into allantoin, a more soluble product, thereby facilitating its excretion [10]. However, in humans and some primates, uricase has become nonfunctional due to pseudogenization, leading to elevated uric acid levels, particularly in individuals with hyperuricemia [8]. Excess uric acid results in the formation of crystals within joints and tissues, causing inflammatory conditions like gout [7][11]. Elevated uric acid levels are also implicated in diseases such as cardiovascular disorders, neurodegenerative diseases, and metabolic syndrome [12][13].

Despite the therapeutic promise of uricase, its immunogenicity presents a major hurdle in clinical applications. As uricase is derived from non-human sources (e.g., bacterial, fungal, or porcine), the human immune system may recognize it as foreign, potentially inducing harmful immune responses, including the formation of neutralizing antibodies that can compromise treatment efficacy [14][15][16][17]. This underscores the critical need to understand the epitopic landscape of uricase, as epitopes—regions on the protein recognized by antibodies—are central to immunogenicity [18][19].

Epitopes can be classified into three categories: linear, which are continuous amino acid sequences; conformational, which arise from the three-dimensional folding of the protein; and quaternary, which involve interactions between different protein chains or subunits. Although much attention has been given to linear and conformational epitopes, quaternary epitopes remain largely underexplored in the context of uricase immunogenicity. This study aims to address this gap by hypothesizing that uricase immunogenicity arises from a combination of linear, conformational, and quaternary epitopes. To investigate these epitopes, we employed an in silico approach, utilizing a range of computational tools and databases to predict potential epitopes on uricase. Through a comprehensive database search, we identified four uricase variants from distinct origins, all of which shared structural similarities despite their sequence variations. These findings suggest a common structural framework that could potentially harbor immunogenic epitopes, thus raising the question of whether the immunogenicity observed across different uricase variants stems from conserved epitopic regions.This study investigates the epitopic landscape of uricase with a focus on identifying linear, conformational, and quaternary epitopes that contribute to its immunogenicity. Through detailed structural alignments and consensus epitope prediction methods, we aim to elucidate factors that could guide the design of less immunogenic uricase variants. By highlighting quaternary epitopes, which involve inter-chain interactions and are understudied in the context of uricase immunogenicity, this research provides insights that could support the development of safer, more effective uricase-based therapeutics for clinical application.

## Materials and Methods

### Data Acquisition

Uricase structures were retrieved from the PDBFlex database [20] (https://pdbflex.org/) using the keyword "uricase." PDBFlex collates crystal structures with a minimum of 95% sequence identity, allowing for the examination of structural variations. These structures were analyzed and visualized using UCSF Chimera ver 1.15 ((http://www.cgl.ucsf.edu/chimera/) [21], a molecular modeling system widely used for protein analysis. Corresponding FASTA sequences were extracted for further sequence-based analyses.

### Structural Alignment

The structural alignment of uricase variants was performed using the Matchmaker function within UCSF Chimera. This tool utilizes the BLOSUM 62 substitution matrix and the Smith-Waterman algorithm [22] algorithm to superimpose protein structures with high sensitivity, ensuring the best scoring alignments are captured.

### Sequence Property Analysis

Various B-cell epitope-related sequence properties were predicted using tools from the Immune Epitope Database (IEDB) (https://www.iedb.org/), focusing on the following parameters: B-cell epitopes: hydrophilicity [23], flexibility [24], surface accessibility [25] and antigenicity [26].These properties were assessed to identify regions likely to elicit immune responses.

### Linear B-cell Epitope Prediction

Linear B-cell epitopes were predicted using multiple algorithms to enhance reliability: Bepipred [27][28][29] (threshold: 0.35), Bepipred 2 [30] (threshold: 0.5), as provided by www.iedb.org, and Bepipred 3 [31] (threshold: 0.15), available through the IEDB and HealthTech DTU services.

Ellipro [32] (threshold: 0.5) was also used to predict linear epitopes based on structural data and machine learning techniques.

Each BepiPred version integrates different computational approaches, ranging from hidden Markov models (BepiPred-1.0) to advanced machine learning with protein language models (BepiPred-3.0). Consensus epitopes were selected by combining predictions from these tools with physicochemical properties for robust identification of immunogenic sites.

### Conformational B-cell Epitope Prediction

Conformational (discontinuous) epitopes were identified using three complementary tools:

DiscoTope 3.0 [33] (DTU HealthTech) - This algorithm incorporates surface accessibility, residue clustering, and solvent accessibility to locate epitopes based on protein structures.

Ellipro [32] - A structural epitope prediction tool from IEDB that uses machine learning and geometric principles.

SEMA [34] (https://sema.airi.net) - This deep learning-based tool utilizes transfer learning to predict conformational epitopes, integrating both sequence and tertiary structure information (threshold: 1.1).

###  Quaternary Epitope Localization

To localize quaternary epitopes, the conformational epitopes on one chain of the uricase structure were first identified. Residues within a 6-angstrom radius of these epitopes on adjacent chains were then selected as potential quaternary epitopes. This proximity-based method allows for the identification of epitopes formed through inter-chain interactions, which are critical for understanding the immunogenicity of protein complexes.

### Interaction Network Analysis

To further explore the interaction network of quaternary epitopes, residue interaction networks were constructed based on a 6-angstrom cutoff distance between Cα atoms in the protein structures. The RINalyzer [35] plugin for Cytoscape 3.9.1 [36]was used to generate residue interaction networks from the Protein Data Bank (PDB) structures.

RINalyzer established a connection between Cytoscape and UCSF Chimera [21], allowing for the extraction and visualization of interaction data. Interface residue subnetworks, defined by residues from different chains within 6-angstrom proximity, were isolated for further analysis. These subnetworks specifically included residues involved in conformational epitope formation, enhancing the identification of quaternary epitopes.

## Results

**Figure-1:**
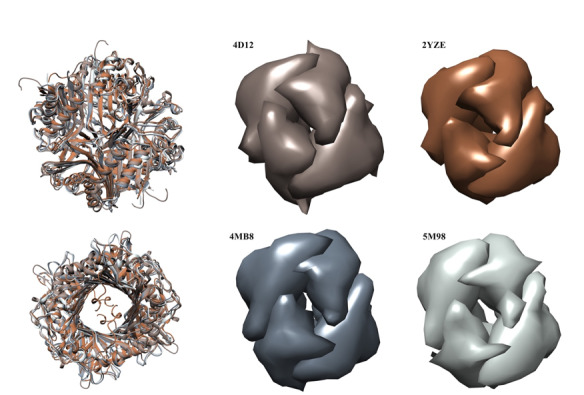
Figure 1. The conformational confluence of the studied uricases is depicted through structural alignment. Four enzyme variants are distinctly color-coded for clarity. The left panel illustrates the structural alignment of these four uricases, offering two distinct views for comprehensive analysis. The middle and right panels present schematic representations of these enzymes, providing a visual overview of their structural features. These images were meticulously crafted using UCSF Chimera version 1.15.

**Table:** Table1. Four Representative Structures for Uricases

**PDB ID**	Cluster Members ^**^	**Organism**
**4D12**	102	Aspergillus flavus
**2YZE**	51	Arthrobacter globiformis
**4MB8**	3	unclassified Mammalia
**5M98**	15	Danio rerio

### Structural Analysis of Uricase Variants

Using the PDBFlex database, we identified four representative uricase structures from distinct organisms. These structures (Table-1) were selected to minimize redundancy, as PDBFlex clusters structures with over 95% sequence identity. The chosen uricase enzymes were sourced from Aspergillus flavus, Arthrobacter globiformis, Mammalia, and Danio rerio, representing a diverse range of uricases for in-depth analysis.

### Structural Alignment and Conservation

Despite variations in amino acid sequences, the overall structural topology of the uricase enzymes remained highly conserved. The uricases adopted a homotetrameric structure, forming a tightly enclosed tunnel through which uric acid catalysis occurs (Figure-1). Structural alignment using UCSF Chimera revealed significant conformational similarity across the four variants.

### Physicochemical Properties of Uricase Sequences

**Figure-2:**
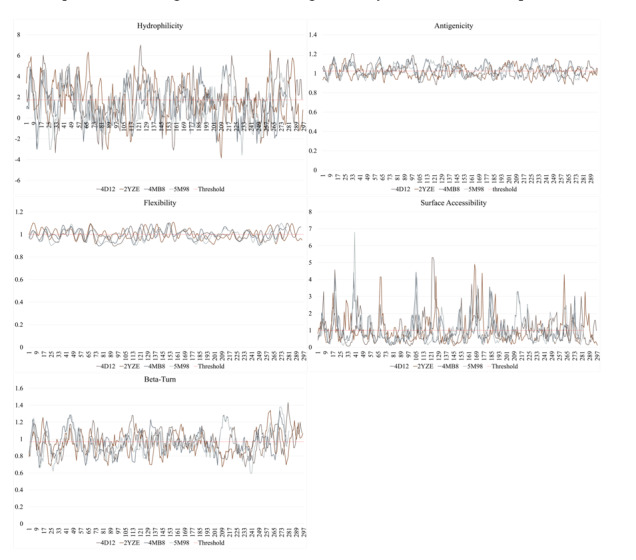
Figure 2. Physicochemical properties of four studied enzymes. Each graph contains one property related to four sequences. The X-axes in all graphs are amino acid numbers and the Y-axes are related scores. The horizontal red line defines the threshold.

To investigate immunogenic determinants, we analyzed the physicochemical properties of the uricase sequences, focusing on parameters such as antigenicity, beta-turn propensity, hydrophilicity, flexibility, and surface accessibility. These properties play a crucial role in B-cell epitope localization. As shown in Figure-2, the profiles exhibited consistent fluctuations across all four sequences, reflecting similar trends in regions likely to elicit immune responses.

### Consensus Epitope Identification

**Figure-3:**
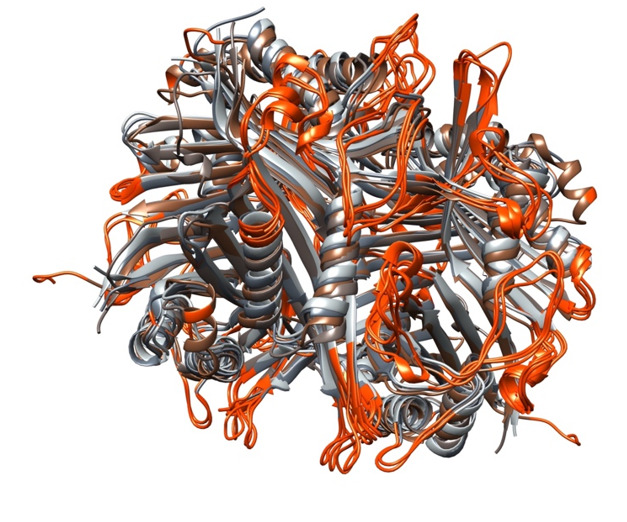
Figure 3. Location of consensus B-cell epitopes on the structural alignment of four uricase enzymes. The structural alignment of four uricase enzymes highlights the location of consensus B-cell epitopes. The enzymes are represented by ribbons, with colors resembling those in Figure 1. The B-cell epitopes are distinguished by their red coloration. Despite differences in the amino acid sequences, the overlapping positions of the epitopes are clearly evident in the structural alignment. This observation demonstrates the common location of these epitopes among the different uricase enzymes despite variations in their amino acid sequences.

Linear and conformational epitopes were predicted using multiple computational algorithms, including BepiPred (versions 1, 2, and 3) and Ellipro. Consensus epitopes were defined as regions receiving positive predictions from at least two algorithms. Table-2 lists the consensus epitopes for each uricase variant. Uricases show consistent topologies, despite variations in amino acid arrangements (Figure-1). Through the location of consensus B-cell epitopes in the enzyme structures and the concurrent alignment of these structures, it was observed that B-cell epitopes are presented in analogous topologies on the enzyme surfaces (Figure-3). This is also evident in Table-1, where the positions of epitopes are approximately similar despite variations in sequence of peptides.

### Quaternary Epitope Characterization

A novel aspect of this study was the characterization of quaternary epitopes formed through inter-chain interactions within the uricase complexes. Using a network-based approach, residues located within 6-angstrom proximity across different chains were identified as potential quaternary epitopes. The residue interaction networks were constructed using RINalyzer in Cytoscape, with a focus on interface residues that form part of the immunogenic landscape (Table-3).

**Figure-4:**
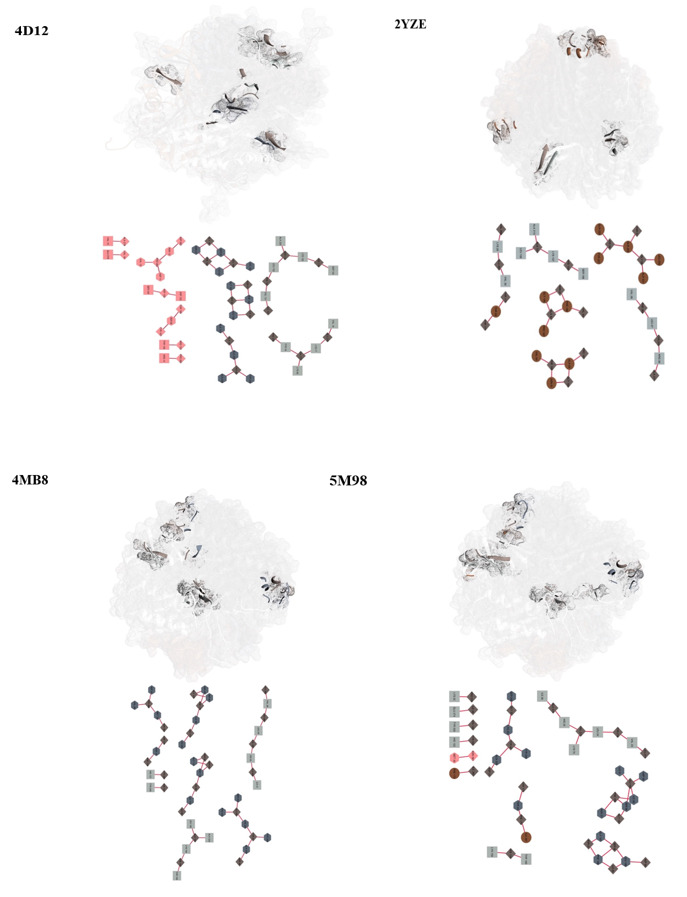
Figure 4. The location of quaternary epitopes on the surface of uricases. The surface location of quaternary epitopes on uricases is illustrated in the left panel. The interaction network of epitopic residues is depicted, where the entire structure is transparent except for the quaternary epitopes. These quaternary epitopes consist of residues originating from at least two different chains. In the network representation, each node corresponds to an amino acid, with diamond nodes representing residues from chain A, ellipses denoting chain B, hexagons indicating chain C, and rectangles representing chain D. The nodes and chains are color-coded differently to facilitate visual differentiation.

The construction of each network involved a systematic process of selecting predefined epitopes, identifying residues within a 6-angstrom radius around them, and extracting subnetworks comprising interface residues. Through the integration of Chimera and Cytoscape, the selected clusters (Table-3) were efficiently visualized and mapped to the relevant molecular structure (Figure-4).

Consequently, each structure encompasses multiple epitopic regions that arise from the folding of multiple chains. In contrast, the monomers do not exhibit such epitopic regions.

The identification of quaternary epitopes adds an important dimension to understanding the immunogenic properties of uricase, particularly in the context of protein-protein interactions. These findings could inform the development of next-generation therapeutic uricases with reduced immunogenicity.

## Discussion

**Table:** Table-2. The Consensus Epitopes Defined for each Uricase

**4D12**		**2YZE** **[1] ** [MR2]		**4MB8**		**5M98**	
**start & end**	Sequence	start & end	Sequence	start & end	Sequence	start & end	Sequence
**18-28**	VHKDEKTGVQT	32-37	RNTARH	33-37	RDGKY	26-35	IRREGNHHHI
**41-54**	EIETSYTKADNSVI	51-64	DFEAAHTAGDNAHV	50-67	TLSSKKDYLHGDNSDIIP	47-60	KTRKDYLTGDNSDI
**89-93**	EKYNH	98-102	EGFDW	100-1006	SSFNHVI	95-101	TAFNHVT
**106-123**	WTRMDIDGKPHPHSFIRD	117-124	RINDHDHA	121-130	EKNGVKHVHA	115-124	LEKNGVEHNH
**160-175**	WGFLRDEYTTLKETWD	164-177	HGFPRDKYTTLQET	169-186	GFEGFIKDQFTTLPEVKD	162-180	QTGFEGFLRDRFTTLTDAK
**195**	Q	196	E	202	G	198-	N
				225-231	PYDKGEY	218-225	GPYDRGEY
**260-284**	IDLSWHKGLQNTGKNAEVFAPQSDP	255--272	VDLQPFGQDNPNEVFYAA	266-288	FNIDMSKMGLINKEEVLLPLDNP	264-281	MTKIGLSNKDEVYLPLDN

**Table:** Table3. List of Quaternary Epitopes Defined on the Surface of each Structure

**Structure**	**Cluster of Quaternary Epitopes**
	TYR.69.D, LYS.17.D, VAL.18.D, GLU.276.A, VAL.277.A, PHE.278.A
	VAL.151.D, LEU.152.D, LYS.153.D, SER.154.D, ALA.220.D, HIS.118.A, SER.119.A, PHE.120.A, ILE.121.A
**4D12**	ALA.225, GLN.228, ALA.229, TYR.232.C, TYR.46.A, ALA.49.A
	GLY.161.C, PHE.162.C, LEU.163.C, ASN.51.A, SER.52.C, ILE.154.C
	ASN.51.C, SER.52.C, ILE.54.C, VSL.55.C, GLY.161.A, PHE.162.A, LEU.163.A
	ARG.26.D, LEU.27.D, VAL.28.D, PHE.269.A, TYR.270.A, ALA.271.A
	LEU.220.B, ALA.221.B, GLN.223.B, GLN.224.B, TYR.227.B, HIS.56.A, THRR.57.A, GLY.59.A
	LEU.156.D, LYS.157.D, SER.158.D, ALA.215.D, HIS.123.A, ALA.124.A
**2YZE**	LYS.29.D, VAL.30.D, GLU.267.A, VAL.268.A
	THR.69.B, TYR.171.A, THR.172.A
	GLY.165.B, PHE.166.B, PRO.167.B, ASN.61.A, ALA.62.A, VAL.64.A
	ASN.61.B, ALA.62.B, VAL.64.B, GLY.165.A, PHE.166.A, PRO.167.A
	PRO.233.C, SER.234.C, GLN.236.C, LYS.237.C, TYR.240.C, ASP.56.A, TYR.57.A, GLY.60.A
	VAL.28.D, LEU.29.D, HIS.30.D, ILE.31.D, GLU..280.A, VAL.281.A, LEU.282.A, LEU.283.A
	ASN.62.C, SER.63.C, ILE.65.C, ILE.66.C, GLY.172.A, PHE.173.A, ILE.174.A
**4MB8**	GLY.172.C, PHE.173.C, ILE.174.C, ASN.62.A, SER.63.A, ILE.65.A, ILE.66.A
	ASP.24.C, MET.25.C, VAL.26.C, ASN.287.A, PRO.288.A
	THR.70.C, PHE.178.A, THR.179.A
	HIS.127.D, TYR.226.D, HIS.127.A, TYR.226.A
	LEU.163.D, LYS.164.D, THR.165.D, ALA.223.D, HIS.129.A, ALA.130.A
	GLY.167.C, PHE.168.C, LEU.169.C, ASN.57.A, SER.58.A, ASP.59.A, ILE.60.A
	ASN.57.C, SER.58.C, ASP.59.C, ILE.60.C, ILEU.61.C, GLY.167.A, PHE.168.A, LEU.169.A
	VAL.23.D, LEU.24.D, HIS.25.D, ILE.26.D, LYS.75.D, GLU.274.A, VAL.275.A, TYR.276.A, LEU.277.A
	SER.228.C, GLN.230.C, LYS.231.C, TYR.234.C, ASP.51.A, TYR.52.A, GLY.55.A
	THR.65.C, ILE.267.B, THR.174.A, PHE.173.A
**5M98**	LYS.159.D, THR.160.D, HIS.124.A
	LYS.22.D, PRO.278.A
	GLU.274.D, ILEU.26.A
	TYR.220.D, HIS.122.A
	HIS.122.D, TYR.220.A
	PHE.173.B, ILE.276.A

Therapeutic proteins, such as protein-based drugs, are biologically active agents designed to treat various medical conditions by mimicking or augmenting the physiological activities of endogenous proteins or targeting proteins involved in pathological processes [37]. Uricase, a key oxidoreductase enzyme, plays a critical role in purine metabolism by catalyzing the conversion of uric acid into the more soluble compound, allantoin, facilitating the excretion of excess purine metabolites [38]. While uricase shows substantial therapeutic potential [39][7][40], its clinical application is often hindered by immunogenic responses in the host due to its non-human origin and the need for repeated administrations [41].

Uricases are found in various sources, including bacteria (e.g., Pseudomonas aeruginosa, Arthrobacter globiformis, Bacillus subtilis), fungi, and mammalian tissues. Commercial uricases, derived from Aspergillus flavus and porcine liver, have encountered significant limitations, such as short half-life, hypersensitivity reactions, and pronounced immunogenicity. To address these challenges, the development of polyethylene glycol-modified uricase (PEG-uricase or Pegloticase) has improved its solubility and half-life, while attempting to reduce immunogenicity [42][43][44]. However, hypersensitivity reactions remain prevalent in both bacterial and mammalian uricases [44], with side effects ranging from gastrointestinal disturbances to fever and nausea [45][46].

The immunogenicity of uricase is thought to arise from specific epitopes, particularly conformational and quaternary epitopes, formed through the complex folding of the enzyme and its multimeric assembly. Understanding these structural elements of uricase that interact with the immune system is crucial not only for designing safer uricase therapies but also for informing broader protein engineering approaches. Therefore, detailed investigations into the structural elements of uricase that interact with the immune system are crucial to understanding and mitigating immune responses. This study focuses on identifying the epitopic determinants that contribute to uricase immunogenicity, with particular emphasis on quaternary epitopes, which remain largely underexplored.

Antibody recognition, a key component of the humoral immune response, is mediated by B-cells that identify specific regions of antigens known as epitopes [47]. Accurate identification of these epitopes is essential for vaccine design, diagnostic tools, and therapeutic antibody development [48][49][50]. While experimental methods for epitope mapping—such as X-ray crystallography, pepscan, and phage display—are effective, they are resource-intensive and often imprecise in pinpointing epitope locations [51]. In contrast, in silico approaches provide a faster, cost-effective means of epitope identification, employing various algorithms to predict linear, conformational, and quaternary epitopes based on sequence and structural data [52].

In this study, four structurally similar uricase enzymes were analyzed, revealing conserved topological features across diverse sources. This observation aligns with previous reports of conserved structural motifs within urate oxidase [53][54][17]. Through in silico analysis, we identified key regions likely responsible for immunogenic responses, thereby offering potential targets for modifying the uricase structure to reduce its immunogenicity. Our study focused on both linear and conformational epitopes, with particular emphasis on the novel identification of quaternary epitopes—formed by inter-chain interactions within the uricase multimeric structure. Conformational epitopes, consisting of spatially adjacent amino acid residues, were identified using advanced computational algorithms such as DiscoTope, Ellipro, and SEMA. Although conformational epitope prediction is well-established, few tools accurately predict quaternary epitopes, particularly in proteins with complex multimeric structures [55]. By leveraging interaction networks and structural modeling, we successfully mapped quaternary epitopes—critical regions that span multiple chains within the uricase structure. Our findings suggest that these epitopes could play a pivotal role in the immune response to uricase, as they likely provide unique interaction points for antibodies that are specific to the enzyme’s multimeric configuration.

The potential clinical implications of these quaternary epitopes are significant. In particular, quaternary epitopes in uricase may influence antibody-mediated immune responses due to their unique structural characteristics, which could enable new avenues for designing uricase variants with reduced immunogenicity.

Quaternary epitopes have been previously characterized in proteins composed of multiple chains, such as glycoproteins found in various viruses [56][57]. These viral epitopes are often critical in antibody-mediated neutralization, as demonstrated in studies on glycoproteins of several viral pathogens [58][59][60][61]. Antibodies that specifically target quaternary epitopes play a crucial role in neutralizing viruses [61][62][59], highlighting the potential importance of similar epitopes in the immunogenicity of therapeutic proteins like uricase. Drawing from this viral glycoprotein analogy, the quaternary epitopes observed in uricase may elicit a robust antibody response due to their unique inter-chain interactions, underscoring the potential of targeting these epitopes in future immunogenicity-reducing strategies.

While this study has advanced our understanding of uricase epitopes, it is important to note its limitations. One primary limitation is the study’s focus on B-cell epitope prediction, specifically linear, conformational, and quaternary epitopes, without the inclusion of T-cell epitope analysis. The role of T-cell epitopes in uricase immunogenicity, though previously documented [53][17][14], remains an essential aspect for future studies to address, as T-cell responses are critical to the immunogenic profile of therapeutic proteins. Exploring T-cell epitopes could yield further insights into the overall immune response to uricase and enhance therapeutic designs.

Additionally, while computational predictions offer valuable insights, they require experimental validation to confirm the immunogenicity of the predicted epitopes. Follow-up studies utilizing techniques such as enzyme-linked immunosorbent assays (ELISA) and other immunoassays will be essential for verifying the immune recognition and relevance of these epitopes. This study serves as a stepping stone, and further experimental work is needed to fully elucidate the immunogenic profile of uricase.

Herein we emphasize the importance of quaternary epitopes as critical determinants in uricase immunogenicity. By integrating computational tools, we provide a comprehensive analysis of uricase’s epitopic landscape, highlighting regions that could be targeted to design less immunogenic uricase variants.

## Conclusion

This study represents a significant step forward in understanding the immunogenicity of uricase by integrating computational tools to predict B cell epitopes, both linear and conformational, across uricase sequences. The application of multiple prediction algorithms, alongside analyses of physicochemical properties, provided robust evidence for epitope localization and its relationship to structural characteristics. Importantly, our research highlights the previously underexplored role of quaternary epitopes, which are formed by interactions across multiple polypeptide chains, thus offering a novel perspective on uricase’s immunogenic potential.

The identification of these epitopes offers critical insights that could inform the design of less immunogenic uricase variants, thereby improving therapeutic applications. Although our methodology involves multiple tools and steps, there is scope for further development to streamline and automate the process. Future studies should aim to experimentally validate these computational predictions and assess the clinical relevance of quaternary epitopes in the context of immune responses. Such advancements will be crucial for the development of optimized uricase therapies with minimized immunogenicity.

## Conflict of Interest

The authors declare no competing interests.
